# Cone‐Beam Computed Tomography of Osteogenesis Imperfecta Types III and IV: Three‐Dimensional Evaluation of Craniofacial Features and Upper Airways

**DOI:** 10.1002/jbm4.10124

**Published:** 2019-02-07

**Authors:** Natalie Reznikov, Didem Dagdeviren, Faleh Tamimi, Francis Glorieux, Frank Rauch, Jean‐Marc Retrouvey

**Affiliations:** ^1^ Faculty of Dentistry McGill University Montreal Canada; ^2^ Shriners Hospitals for Children–Canada Montreal Canada

**Keywords:** OSTEOGENESIS IMPERFECTA, CRANIOFACIAL DEFORMITIES, CONE‐BEAM COMPUTED TOMOGRAPHY, AIRWAYS, CRANIAL BASE

## Abstract

This cross‐sectional study investigated the natural history of craniofacial deformities in osteogenesis imperfecta (OI) and determined the impact of three‐dimensional (3D) analysis on diagnosis and treatment planning in orthodontics and orthognathic surgery in comparison to conventional two‐dimensional (2D) cephalometric examination. 3D images of the craniofacial complex were acquired during 1 calendar year using cone‐beam computed tomography (CBCT) from a cohort of 41 individuals (aged 11 to 35 years; 28 females) with OI type III (*n* = 13) or IV (*n* = 28). 3D evaluation of the craniocervical junction and upper airways was conducted using InVivo^TM^. 2D lateral cephalogram was constructed, traced, and examined using the University of Western Ontario analysis (Dolphin^TM^). Quantitative and qualitative parameters were compared between OI type III and type IV groups (unpaired *t* test) and the unaffected population (*Z*‐score). 3D evaluation revealed a high prevalence of craniocervical abnormalities, craniofacial asymmetries, and nasal septum deviation in both OI groups. Mean airway dimensions were comparable to the non‐affected population norms, except for 5 individuals who had insufficient airway dimensions. In 2D, the maxilla was retrognathic and hypoplastic, and the mandibular position was convergent with respect to the face, resulting in mandibular prognathism and face height reduction. The 2D trends were more pronounced in OI type III, whereas the 3D craniocervical and airway abnormalities were common in both types. This study illustrates the prevalence of craniofacial and airway anomalies in OI that occur along with facial deformities are not associated with postcranial phenotype and OI type, are apparent only in 3D evaluation, and are likely to influence treatment strategy. For OI patients, a team effort involving a dentist, orthodontist, neurologist, and ear‐nose‐throat (ENT) practitioner is recommended for successful management of craniofacial deformities. © 2018 The Authors *JBMR Plus* published by Wiley Periodicals, Inc. on behalf of American Society for Bone and Mineral Research.

## Introduction

Osteogenesis imperfecta (OI) is a rare genetic disorder that is characterized by frequent bone fractures, short stature, deformities of long bones and spine, craniofacial anomalies, dentinogenesis imperfecta, sclerae discoloration, and joint hyperlaxity.^1^, ^2^ The traditional classification of OI distinguishes four types:^1^, ^2^, ^3^ type I is mild, type II is perinatally lethal, type III is severe/deforming, and type IV is moderate‐severe. The clinical assignment of OI type III or OI type IV is based on patient mobility and stature.^4^ Other types of OI are rare.^1^, ^2^ In 90% of individuals having a clinical diagnosis of OI, the disease can be explained by pathologic variants in *COL1A1* and *COL1A2*, the genes encoding collagen type I alpha chains.^5^ However, of the patients with moderate and severe forms of OI, 20% have mutations other than *COL1A1* and *COL1A2*. Moreover, different types of mutations (splice, or nonsense mutation, or amino acid substitution) and their localization along the sequence result in diverse manifestations of the disease,^5^ with some degree of bone fragility being a characteristic common denominator for all OI types.

Craniofacial and dentoalveolar abnormalities are present in mild, moderate, and severe types of OI. Cephalometric studies have revealed that dentoalveolar structures and the condylar processes are often vertically underdeveloped in OI types III and IV.^6^, ^7^, ^8^, ^9^ Clinically, the hypoplastic maxilla and reduced vertical and transverse dimensions result in a malocclusion, such as bilateral open bite or cross bite, and counterclockwise (overclosing) rotation of the mandible.^9^ The facial appearance of moderate and severe OI is often characterized by frontal bossing, triangular face shape,^3^ and macrocephaly.^10^, ^11^, ^12^ One of the alarming consequences of the skull deformities in OI is the high occurrence of craniocervical junction anomalies^4^, ^13^, ^14^ that are often accompanied by neurological symptoms,^10^ varying from reduced muscular tone to fatal symptoms of brain stem compression and central apnea that require urgent surgical intervention.^13^, ^14^, ^15^, ^16^ The shape of the cranial base also affects the geometry of the upper airways.^17^ Recent research has shown a possible positive correlation present between the cranial base angle value and the upper airways minimal cross‐sectional area in the non‐OI population.^18^ A broader agreement states that the retroposition of both mandible and maxilla reduces the airways cross section.^19^, ^20^, ^21^ Considering the maxilla retroposition^9^ and compromised respiratory function in OI patients,^22^ the assessment of the upper airways in the context of craniofacial deformities is of clinical significance.

This study investigated the impact of three‐dimensional (3D) analysis on diagnosis and treatment planning in orthodontics and orthognathic surgery compared with conventional two‐dimensional (2D) cephalometric examination.

## Materials and Methods

### Study participants

Individuals with a diagnosis of OI type III or IV were recruited through the Brittle Bone Disease (BBD) Consortium (https://www.rarediseasesnetwork.org/cms/BBD) that is composed of several specialized centers across North America (Baltimore, Chicago, Houston, Los Angeles, Montreal, New York, Omaha, Portland, and Washington, DC). This consortium is part of the Rare Disease Clinical Research Network funded by the National Institutes of Health (USA). One of the projects conducted by the BBD consortium is to assess the craniofacial features of moderate to severe OI population (defined as primarily OI types III and IV) by using CBCT acquired at McGill University, Faculty of Dentistry, in Montreal, Canada. Because this methodology requires considerable patient compliance, this project was limited to individuals aged 10 years or older. Informed consent was obtained from the study participants or their guardians, and IRB approval was granted.

The present study included 41 individuals who were diagnosed with OI type III (*n* = 13) or OI Type IV (*n* = 28) based on clinical presentation^23^ (Table 1). These 41 patients were earlier enrolled in a DNA sequence and mutation spectrum study along with other 557 patients who did not participate in the current study of craniofacial deformities.^5^ Of the 13 individuals diagnosed with OI type III, 6 had mutations in *COL1A1*, 5 had mutations in *COL1A2*, and 2 had biallelic mutations in *CRTAP*. Among the 28 participants diagnosed with OI type IV, 15 had mutations in *COL1A1*, 11 had mutations in *COL1A2*, one had a mutation in *WNT1*, and in one individual targeted sequencing of an OI gene panel^5^ did not reveal a disease‐causing mutation. Among the type IV patients, two 13‐year‐old females were homozygous twins carrying a COL1A1 Gly‐to‐Cys substitution mutation in position 845.

**Table 1:** Biological Profile and Clinical Characteristics of the Study Population

	OI total	OI type III	OI type IV	*p* (III versus IV)
*n* (female/male)	41 (28/13)	13 (11/2)	28 (17/11)	0.126^a^
Age (mean ± SD; years)	19.0 ± 7.4	20.6 ± 8.6	18.2 ± 6.8	0.333^b^
Height *Z*‐score (mean ± SD)	−5.2 ± 3.3	−8.8 ± 2.4	−3.6 ± 2.1	<0.001^b^
Weight *Z*‐score (mean ± SD)	−1.3 ± 1.5	−2.5 ± 0.9	−0.8 ± 1.4	<0.001^b^

^a^ The *p* value was calculated using chi‐square test.

^b^ The *p* value was calculated using unpaired *t* test for normally distributed data.

Height/weight correlation coefficient *r* = 0.755.

### Cone‐beam computed tomography (CBCT)

CBCT scans were acquired with a 3D Accuitomo 170 (Morita Inc, Kyoto, Japan) CBCT machine in a 170 mm × 120 mm field‐of‐view and a 250 μm voxel size. The exposure settings for CBCT included a tube voltage of 90 kV and a tube current of 4.5 mA, at 17.5 seconds. Each patient was seated in the CBCT apparatus in such a way that the Frankfurt (auriculo‐orbital) plane coincided with the true horizontal plane to compensate for the postural asymmetry common in OI. A head restraint around the patient's forehead and a chin rest were used to standardize the patient's head position. Image analysis was performed using Anatomage InVivo 5 version 5.4 (InVivo Dental; Anatomage, San Jose, CA, USA) software.

### 3D Craniofacial evaluation

**Figure 1:**
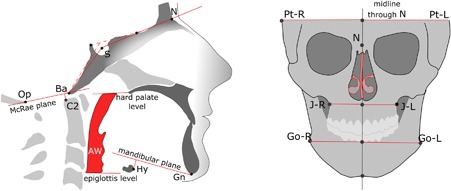
Diagram of the measurements obtained from CBCT volumetric images. Cranial base angle traditional measurement is shown by solid lines and modified measurement is shown by dashed lines. The distance between the apex of the C2 odontoid process is measured to the plane of foramen magnum (negative value = normal; positive value = basilar invagination).

The following parameters were assessed on 3D data sets. The cranial base angle was measured using two techniques:^24^ by the standard method as an angle formed between the nasion (N) – center of pituitary fossa (sella, S) – midpoint on the anterior border of foramen magnum (basion, Ba), and by the method modified for tomography, between the line connecting the floor of anterior cranial fossa to dorsum sellae and the line passing through the clivus. Although the former method is more common in cephalometric analysis, the modified technique benefits from better visibility of the midsagittal structures on a virtual mid‐sagittal tomographic slice. Both cranial base angle measurements provide similar values, but the modified method gives a narrower confidence interval.^24^ The relationship between the cervical spine and cranial base was assessed by measuring the distance between the apex of second cervical vertebra (apC2) and McRae reference plane passing through the foramen magnum, opisthion (Op) – basion (Ba). A positive value was recorded when C2 was above the McRae plane. The upper airways volume and its minimal cross‐section area were measured by 3D semi‐automated grayscale gradient‐based tool using InVivo software. The rostral limit of the airways was set at the level of the posterior border of the hard palate; the caudal limit of the airways was set at the epiglottis level. The airway measurement tool automatically defines the boundaries’ orientation at the defined level as perpendicular to the longitudinal axis of the pharynx. Airway volume and cross section were measured 3 times and the mean value was recorded and rounded to the nearest integer. The height of the hyoid bone with respect to the mandibular plane was measured by constructing a virtual plane through gnathion (Gn) and bilateral gonion (Go) and recording the shortest distance from the anterior border of the hyoid bone (Hy) to that plane. In the transverse dimension, mandibular width (Go‐Go), maxillary width (J‐J), and bitemporal width (Pt‐Pt) values were measured. The transverse measurements were done separately for the right and left landmark with respect to the sagittal mid‐plane passing vertically through nasion, sella turcica, and crista galli. In the case of uncontrolled yaw or tilt of the head, digital alignment was done in InVivo software to achieve symmetrical position. The difference between right and left measurements was recorded and analyzed as an indicator of transverse asymmetries. Nasal septum deformation was evaluated in 2 planes, coronal and axial, and the highest score was assigned. The scoring system was based on the review by Teixeira and colleagues^25^ and was modified for this study as follows: grade 1—nasal septum is not in contact with the conchae at any level, although might be not perfectly straight; grade 2—nasal septum touches the inferior concha but the symmetry of the right and left conchae is roughly preserved; grade 3—nasal septum impinges into the deformed inferior concha; grade 4—nasal septum impinges into, and deforms, the lateral nasal wall. A schematic of landmark positioning and tracing is provided in Fig. 1.

The University of Western Ontario (UWO) cephalometric analysis was selected for the following reasons: it considers the relationships of the dental arches with each other and with the jaws, as well of the jaws with each other and with the cranial structures. As well, it is suitable for orthognathic surgery planning because it incorporates the basic premises of the McNamara analysis,^26^ and it uses sex‐ and age‐adjusted norms for comparison. Automated UWO analysis was conducted using Dolphin cephalometric tracing software. For precise identification of the basion and reproducible positioning of the cranial base template on the lateral 2D cephalogram, the midsagittal virtual slice from 3D CBCT reconstructed volume was superimposed on the 2D cephalometric image.

For quantitative measurements, the mean and standard deviation were calculated; the difference between the type III and type IV OI groups was calculated using an unpaired *t* test, and significance was defined as *p* < 0.05. Mean absolute error was computed to evaluate intraobserver error for 3D measurements. Statistical analysis was performed in Excel 2016 (Microsoft).

## Results

**Figure 2:**
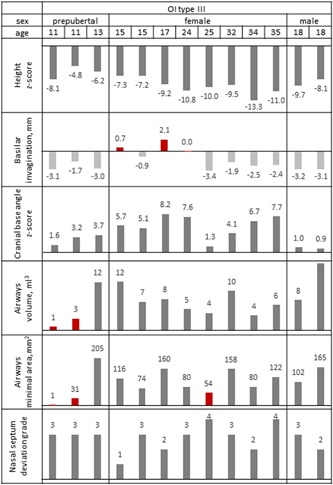
Evaluation of 3D craniofacial parameters in the OI type III group. Red bars indicate clinically concerning values of the basilar invagination and airway volume.

**Figure 3:**
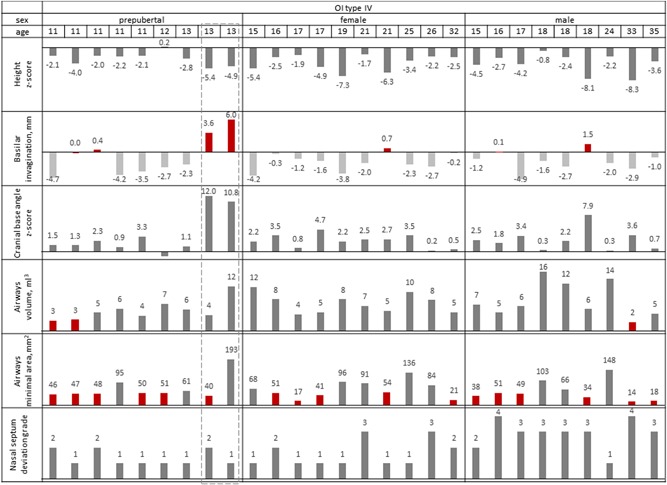
Evaluation of 3D craniofacial parameters in the OI type IV group. Red bars indicate clinically concerning values of the basilar invagination and airways volume. Note the non‐identical parameters of the 2 female twins (13 years old) outlined by a dashed frame. 3D rendering of the twins’ CBCT data is presented in Supplemental Figs. S1 and S2.

**Figure 4:**
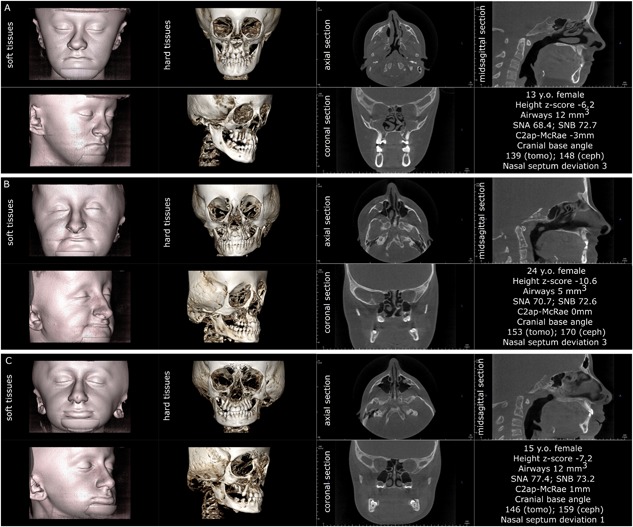
Three individuals affected by OI type III. Note that the triangular face appearance is attributable to the combination of cranial flaring and hypoplastic lower face. All three patients demonstrate the lack of vertical development of the maxilla and insufficient descent of the upper molars, resulting in bilateral open bite. Patients *A* and *B* have severe nasal septum deviation. Patient *A* has platybasia; patients *B* and *C* have both platybasia and basilar invagination (C2ap‐McRae stands for the distance of the apex of the second cervical vertebra above the plane of foramen magnum known as McRae reference line). Patients *A* and *C* have sufficient patency of pharyngeal airways.

**Figure 5:**
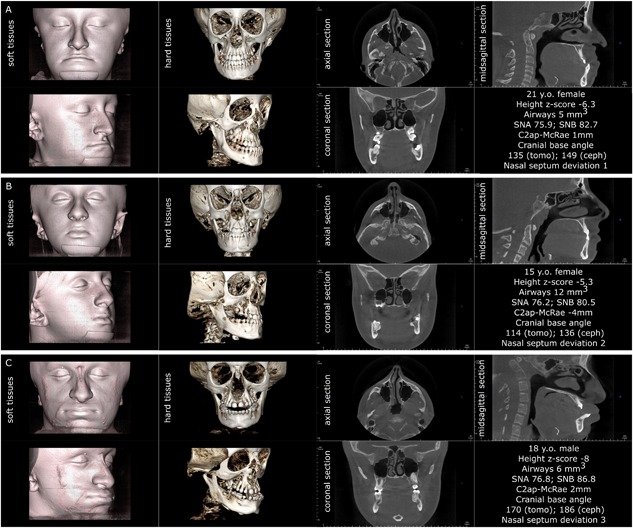
Three individuals affected by OI type IV, assembled according to the visual severity of craniofacial deformities. Note that in OI type IV the triangular face appearance is attributable to the combination of cranial flaring and hypoplastic lower face, similar to OI type III. Patients *B* and *C* demonstrate the lack of vertical development of the maxilla and insufficient descent of the upper molars, resulting in bilateral open bite (patient *B*). Patient *A* has normal nasal septum, patient *B* has moderate nasal septum deviation, and patient *C* has severe nasal septum deviation. Patients *A* and *C* have basilar invagination (C2ap‐McRae represents the distance of the apex of the second cervical vertebra above the plane of foramen magnum known as McRae reference line). Patient *B* has normal craniocervical junction and patient *C* has platybasia.

General assessment in terms of cranial base morphology, airways, nasal septum deviation, and craniofacial asymmetry revealed pronounced diversity among the study participants, both OI type III and type IV. Figs. 2 and 3 illustrate the heterogeneity of the 3D parameters plotted as individual measurements, in patients arranged by age and sex. Comparison of the measured parameters with the height *Z*‐score did not reveal any obvious trend or correlation. OI type IV males generally had more pronounced nasal septum deviation than OI type IV females or OI type IV prepubertal children. As well, in the OI type III group, both adult males had their cranial base angle values closest to the norm. Statistical comparison of the 3D parameters are shown in Figs. 2 and 3 and in Table 2. Examples of broadly varying craniofacial deformities are illustrated in Fig. 4 (OI type III) and Fig. 5 (OI type IV).

**Table 2:** Measurements Obtained From 3D‐Rendered Images

	OI type III (*n* = 13)		OI type IV (*n* = 28)		III versus IV	Non‐OI	
Measurements	Mean value (SD)	Deviants from norm	Mean value (SD)	Deviants from norm	Unpaired *t* test	Non‐OI norm	Ref. no.
Cranial base angle (cephalometry) (°)	152 (15)	9/13	145 (17)	12/28	0.00105	129 (6)	(24)
Cranial base angle (tomography) (°)	140 (15)	10/13	130 (15)	11/28	0.00139	117 (6)	(24)
Basilar invagination (mm)	–1.7 (1.8)	3/13	–1.4 (2.5)	7/28	0.96956	<0	(16)
Airway volume (mL^3^)	7.6 (4.5)	2/13	6.9 (3.4)	3/28	0.59630	6 (1.7)	(32)
Minimal airway cross section (mm^2^)	104 (58)	3/13	65 (42)	17/28	0.01889	120 (61)	(21)
Hyoid height (mm)	8.3 (4.9)	0/13	10.9 (4.5)	0/28	0.11591	15 (3)	(33)
Maxillary asymmetry (mm)	2.2 (1.8)	3/13	1.8 (1.4)	4/28	0.44126	<3	(34)
Mandibular asymmetry (mm)	2.9 (2.3)	4/13	3.1 (2.7)	9/28	0.80826	<3	(34)
Cranial asymmetry (mm)	6.1 (4.1)	10/13	2.4 (2.5)	15/28	0.01713	<3	(34)
Nasal septum deviation (grade)	2.8 (0.8)	12/13	1.9 (1.0)	15/28	0.013061	1	(25)

Mean (standard deviation [SD]) is shown in comparison with historical non‐OI norms from the literature. For the cranial base, the proportion of individuals with deviant measurements was evaluated as exceeding the non‐OI norm plus 2 SD. For the airways, the proportion of individuals with deviant measurements was evaluated as exceeding the non‐OI norm minus 1 SD. Statistically significant differences between OI type III and IV are indicated by a frame. Note that the mean airway volume is normal and the mean craniocervical relationship also appears normal, despite the fact that certain individuals have abnormal values.

Intraobserver (NR) error: cranial base angle (*n* = 2) 3.2/1.6 OI type III and 3.5/2.2 OI type IV; basilar invagination 0.75 mm OI type III and 0.5 mm OI type IV; airway volume 0.6 mL^3^ OI type III and 0.8 mL^3^ OI type IV; airway cross section 10.3 mm^2^ OI type III and 9.3 mm^2^ OI type IV.

This study compared 2D cephalometric measurements of OI type III and IV to sex‐ and age‐adjusted norms. Several significant differences were evident from the findings (Table 3): there was a significant reduction of all vertical facial dimensions (especially lower anterior but also upper anterior) in both OI types in comparison to the control norms. The face axis was inclined upward, leading to a convergent profile (in both OI type III and type IV, being more pronounced in type III). In accordance with this, the relationship between the mandibular plane and anterior cranial base was found generally convergent in both OI types but more significantly so in OI type III. The longitudinal dimensions of the maxilla and mandible were also reduced in both OI types in comparison to the unaffected population, being more so in OI type III. Despite that, Witts appraisal (projection of the anterior borders of the maxillary and mandibular alveolar arches on the occlusal plane) and the ANB angle (the relative position of the maxilla with respect to the mandible) indicated a prominent tendency toward a negative overbite and mandibular prognathism in both OI groups, especially being pronounced in OI type III. Both upper and lower incisors demonstrated anterior proclination and protrusion in OI type III and type IV, apparently as a dentoalveolar compensation for the skeletal discrepancy. However, the proclination and more acute interincisal angle were not as dramatically different from the non‐OI population norm as the aforementioned skeletal parameters. Although nearly all skeletal trends were more severe in OI type III in comparison to type IV, the broad scatter of measured values and high standard deviations resulted in only a few parameters being significantly different between the OI types: facial axis convergence, ANB angle, and facial convexity (Table 3).

**Table 3:** University of Western Ontario (UWO) Cephalometric Analysis of OI Type III and OI Type IV Compared With Nonaffected Population Norms Adjusted for Age and Sex and With Each Other

	OI type III (*n* = 13)		OI type IV (*n* = 28)		III versus IV
Measurements	Mean value (SD)	Deviation from norm (*Z*‐score)	Mean value (SD)	Deviation from norm (*Z*‐score)	Unpaired *t* test
Dental measurements					
Upper incisor axis U1–plane SN (°)	110.1 (11.9)	1.3	107.2 (8.5)	0.8	0.376542
Upper incisor axis U1–plane NA (mm)	4.2 (2.6)	0.0	4.0 (2.3)	–0.1	0.754984
Upper incisor axis U1–plane NA (°)	32.3 (9.5)	1.7	29.2 (8.3)	1.1	0.293717
U1 most labial‐A (perp to FH) (mm)	5.6 (2.4)	0.9	5.5 (1.9)	1.0	0.958701
Interincisal angle (U1‐L1) (°)	126.9 (14.5)	–0.4	125.3 (16.9)	–0.7	0.771651
L1 protrusion (L1‐APo) (mm)	5.4 (3.8)	1.6	3.8 (3.7)	0.6	0.194101
L1‐NB (mm)	4.2 (2.8)	0.1	3.9 (2.7)	–0.1	0.679704
IMPA (L1‐MP) (°)	93.5 (11.0)	–0.2	94.5 (12.0)	–0.1	0.791095
Skeletal measurements					
Anterior facial height (ANS‐Me) (mm)	**50.4 (8.1)**	**−4.2**	**52.8 (11.4)**	**−3.7**	0.490627
Upper face height (N‐ANS) (mm)	45.8 (5.9)	−1.7	45.8 (10.3)	−1.7	0.987286
UFH:LFH, upper (N‐ANS/N‐Gn) (%)	46.8 (6.1)	1.8	45.7 (3.2)	0.7	0.455578
UFH:LFH, lower (ANS‐Gn/N‐Gn) (%)	53.2 (6.1)	–1.8	54.3 (3.2)	–0.7	0.455578
Facial axis angle (Ba‐Na^Pt‐Gn) (°)	**19.0 (8.6)**	**4.7**	**8.8 (7.7)**	**2.2**	0.000487
Facial angle (FH‐NPo) (°)	**97.4 (6.3)**	**3.0**	93.7 (4.7)	1.7	0.043593
FMA (MP‐FH) (°)	**14.0 (7.9)**	**−2.3**	17.9 (5.6)	−1.4	0.075268
MP‐SN (°)	29.5 (6.0)	−0.6	32.9 (5.9)	0.0	0.091869
SNA (°)	77.8 (5.3)	−1.2	78.0 (5.4)	−1.1	0.913099
SNB (°)	81.2 (7.1)	0.1	78.3 (6.1)	−0.8	0.196701
ANB (°)	**–3.3 (4.6)**	**−3.3**	−0.3 (4.1)	−1.3	0.041717
Convexity (A‐NPo) (mm)	**–3.5 (4.5)**	**−2.3**	−0.6 (3.6)	−0.8	0.029255
Maxillary skeletal (A‐Na Perp) (mm)	2.8 (2.5)	0.7	2.6 (3.6)	0.7	0.871007
Mand. skeletal (Pg‐Na Perp) (mm)	**11.5 (10.0)**	**2.9**	5.9 (7.9)	1.9	0.058009
Wits appraisal (mm)	**–6.8 (6.6)**	**−5.8**	**−4.6 (5.8)**	**−3.6**	0.277141
Maxillary length (Co‐A) (mm)	**70.5 (3.4)**	**−3.9**	**70.3 (15.0)**	**−3.9**	0.963111
Mandibular length (Co‐Gn) (mm)	**99.5 (12.3)**	**−5.5**	**98.5 (21.0)**	**−5.9**	0.880644
Soft tissue measurements					
Upper incisor U1 exposure, lips at rest (mm)	2.9 (2.4)	−0.3	3.6 (3.2)	0.2	0.50082
Lower lip to E‐plane (mm)	0.0 (6.1)	0.8	–0.3 (3.7)	0.7	0.855558
Nasolabial angle (Col‐Sn‐UL) (°)	93.6 (26.1)	−1.1	96.1 (22.2)	−0.7	0.758023
Upper lip thickness at A point (mm)	19.1 (2.7)	0.8	17.7 (3.6)	0.2	0.206317
Upper lip thickness at vermillion border (mm)	16.3 (4.5)	0.9	15.4 (4.4)	0.4	0.56292

Mean values are compared using an unpaired *t* test. Mean values deviating from the norm by more than 2 SD are highlighted in bold. Statistically significant differences between OI type III and OI type IV values are highlighted by a black frame.

Of note, the homozygous twins did not demonstrate identical craniofacial parameters, despite having a similar facial pattern (Supplemental Figs. S1 and S2)

**Figure 6:**
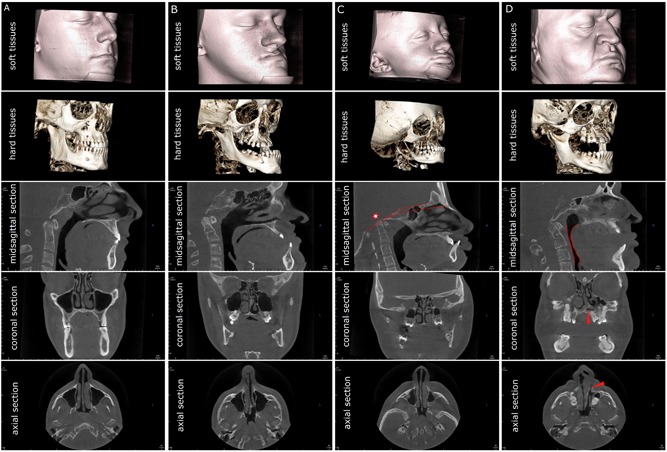
Comparison of the craniofacial parameters in OI type III and type IV patients and their implications for orthodontic treatment planning. Patient *A* (24‐year‐old male, OI type IV) has normal cranial base morphology (118°), normal airways geometry (volume 14 mL^3^), nasal septum deviation grade 1, and a nearly normal occlusion. Patients *B* (18‐year‐old male, OI type III), *C* (17‐year‐old female, OI type III), and *D* (35‐year‐old male, OI type IV) are skeletally mature and have prominent mandibular prognathism, which makes them potential candidates for orthognathic surgery. Patients *B* and *C* have acceptable airway volume of 17 (patient *B*) and 9 (patient *C*) mL^3^. However, the presence of the craniocervical anomaly (platybasia indicated by red lines and basilar invagination indicated by red asterisk) in patient *C* warrants a referral to a neurologist before correction of occlusion. Patient *D* has a normal cranial base morphology (118°), extreme deviation of the nasal septum (grade 4, arrowhead), and low pharyngeal airway volume (4 mL^3^) highlighted in red. Planning of orthodontic treatment for patient *D* should be preceded by an ENT referral for the normalization of breathing pattern and perhaps by posture correction.

In addition, the 3D observations revealed that the upper molar were not descending normally and that the maxillary descent expected with a normal growth pattern was stunted, especially in OI type III patients (Figs. 4, 5, 6). Most of the OI type III patients (12 of 13) and the majority of the OI type IV patients (17 of 28) had noticeable flaring of the calvarial bones (the portion visible in the CBCT field of view) (Figs. 4, 5, 6 and Supplemental Figs. S1 and S2).

Individual analyses for the 41 individuals enrolled in the study are available in Supplemental Tables S1 and S2.

## Discussion

### Cranial base and airway evaluation

The cranial base constitutes the boundary between the neurocranium (braincase) and the viscerocranium (face). The anterior portion of the cranial base angle—the floor of the anterior cranial fossa—significantly defines the morphology of the midface in the sagittal plane;^27^ the posterior portion of the cranial base angle—the floor of the posterior cranial fossa—forms the boundary of the pharynx. The cranial base width defines the mandibular width and, by extension, the geometry of the upper airways.^21^ Being the support structure of the brain and the brain stem, cranial base morphology also defines neurological status and vital functions such as respiration. Considering the high incidence of cranial base anomalies in the OI population, 3D imaging of the head is justified not only for precise planning of orthodontic treatment but also in part for an appropriate referral to a neurologist or otorhinolaryngologist. For example, 3D imaging provides information on the craniocervical junction morphology and airway geometry, these being necessary for differential diagnosis of central sleep apnea (CSA) and obstructive sleep apnea (OSA). In the case of OSA, volumetric imaging is a valuable tool for identification of the level and position of obstruction (eg, nasal, upper pharyngeal, low pharyngeal, or a combination thereof). This study revealed that despite the statistically normal mean airway dimensions in OI patients, some individuals have abnormally low volume and cross‐sectional area of pharyngeal airways. Nasal septum deviation is common in OI, especially in type III, and in males affected by OI type IV—these findings merit screening of the upper airway patency in OI patients. Craniofacial evaluation in 3D in the OI population is likely to result in an amendment or deferment of orthodontic/orthognathic treatment. The following examples of OI type III and IV patients (Fig. 6) illustrate how 3D imaging may raise alarming issues of craniocervical abnormality or upper airway obstruction that requires specialist referral before correction of occlusion. Moreover, the lack of correlation between the prevalence of craniocervical anomalies and airways anomalies and the OI type suggests that 3D‐aided diagnosis and treatment planning are essential. Finally, the notion of clinical significance inferred from 3D evaluation of the craniofacial complex is that the assigned type of OI provides little guidance for treatment without a reliable volumetric imaging method.

### Cephalometric parameters and their analysis in OI

The difficulties in anatomical landmark identification and superimposition of asymmetrical craniofacial structures arise in OI types III and IV, where anatomical relations are often atypical. First, the reference structure used in conventional cephalometric analyses should be the true cranial base angle Ba–S–N, rather than the saddle angle articulare (Ar)–S–N. Although the two angles can be used interchangeably in a person with no severe cranial deformities,^28^ in the case of OI individuals, the discrepancy is such that utilization of Ar–S–N would result in underestimation of the cranial base angle by 20° to 30° and would affect all related measurements. Second, the peculiar shape of the cranial base in OI makes automated template matching in cephalometric software difficult. The utility of 3D CBCT imaging is such that the midsagittal slice with the clearly visible cranial base structures can be used as guidance for tracing and template matching on the virtual 2D cephalometric image.

**Figure 7:**
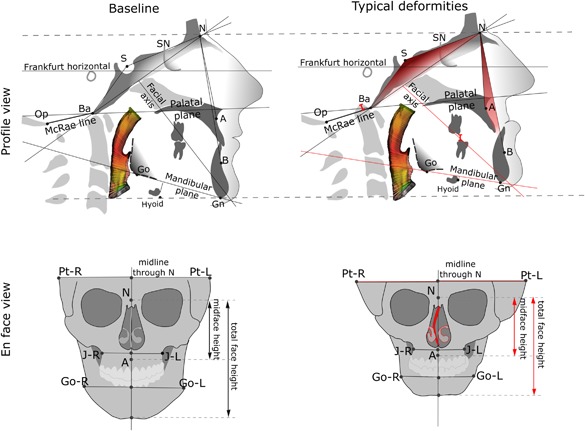
Schematic illustration of the typical craniofacial deformation pattern observed in severe OI: (left) normal craniofacial parameters; (right) generalized trend of craniofacial deformities in severe OI. The more obtuse cranial base angle, the lower ANB angle, the reduced facial height, the overclosed mandible, the basilar invagination, the nasal septum deviation, and the cranial asymmetry in OI are indicated in red. Airway color mapping represents the variability of the cross‐sectional area.

The results of the UWO analysis of the 41 OI patients are concordant with previous studies. As reported previously,^11^, ^29^ the occurrence of class III malocclusion in severe‐deforming OI patients exceeds 60%. The etiology is usually a combination of a hypoplastic maxilla resulting in a negative overjet and deep overbite from the overclosure of the mandible. The hypoplastic maxilla and the overclosed mandible result in an overall reduction of the vertical dimension of the face. This is also in agreement with a lateral cephalometric study^9^ underscoring that the severity of mandibular prognathism is associated with the lack of vertical development of alveolar bone. The severity of mandibular prognathism in OI (attributed to the mandibular counterclockwise, or closing hinge rotation) has been reported to be associated with an increased cranial base angle.^9^ This is in contrast to unaffected cohorts, in whom an increased cranial base angle is associated with a more retrognathic position of the mandible.^30^, ^31^ The combination of a hypoplastic mandible and maxilla and an increase in calvarial width may lead to the visual impression of a triangular face that is frequently reported as a feature of severe OI. The general trend of craniofacial deformities in moderate‐to‐severe OI is given in Fig. 7, as a graphic summary.

## Conclusions

This cross‐sectional natural history study of craniofacial features in OI types III and IV performed using 3D CBCT imaging revealed a high occurrence of potentially life‐threatening abnormalities at the craniocervical junction. This includes anomalies such as basilar invagination (10 of 41 patients) and platybasia (18 of 41 patients), both with no predilection to a particular OI type. Moreover, high heterogeneity of the craniofacial deformities, regardless of the OI type, makes it difficult to predict which OI patients are at risk of developing airway obstruction. The normal mean group values of the airway volume and cross section do not reflect the low individual values in certain patients. Nasal septum deviation was severe to extreme in most OI type III and in males with OI type IV. Cephalometric analysis revealed shortening of all the vertical and sagittal face dimensions in both type III and type IV groups and a strong tendency toward class III malocclusion in OI type III. Even in the cases of prominent malocclusion, comprehensive orthodontic treatment planning should be conducted with the participation of an ear‐nose‐throat (ENT) practitioner and neurologist. Overall, the abnormalities of the craniocervical junction and the upper airways are not associated with the postcranial phenotype and the OI type, and are best evaluated using 3D imaging.

## Disclosures

All authors state that they have no conflicts of interest.

## Supporting information

Supporting Table S1.

Supporting Table S2.

Supporting Figures S1–S2.
