# Supplementary material for: Cone‐Beam Computed Tomography of Osteogenesis Imperfecta Types III and IV: Three‐Dimensional Evaluation of Craniofacial Features and Upper Airways
Source: JBMR Plus. 2019 Feb 7;3(6):e10124. doi: 10.1002/jbm4.10124 (PMC6636768; doi:10.1002/jbm4.10124)
Supplement: Supplementary file 3 — Supporting Figures S1–S2. [file JBM4-3-na-s003.docx]

Cone-Beam Computed Tomography of Osteogenesis Imperfecta Types III and IV: Three-Dimensional Evaluation of Craniofacial Features and Upper Airways

Supplemental Information

Figures S1 and S2 illustrate craniofacial deformities in 13-year old female homozygous twins.

Tables S1 and S2 contain individual UWO cephalometric analyses for all patients enrolled in this study.

Table S1. Individual cephalometric measurements of 13 OI type III patients.

Table S2. Individual cephalometric measurements of 28 OI type IV patients.


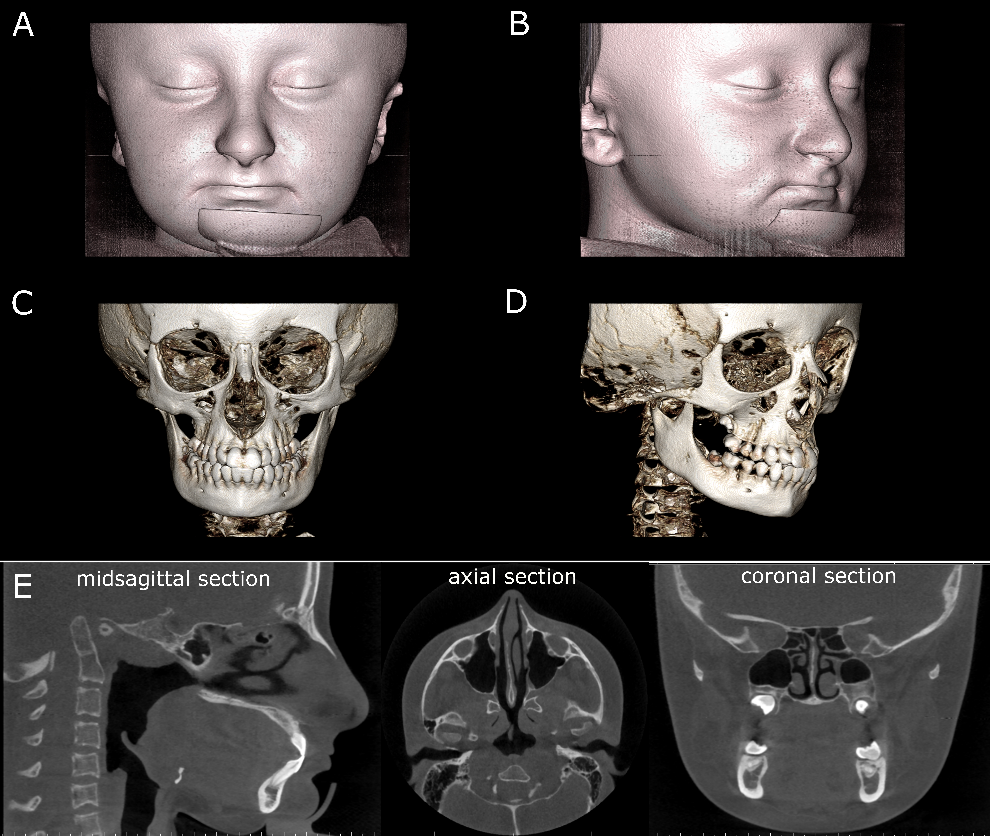
Figure S1. Twin G. has extreme flattening of cranial base and alarming basilar impression. Nasal septum and airways volume are normal.


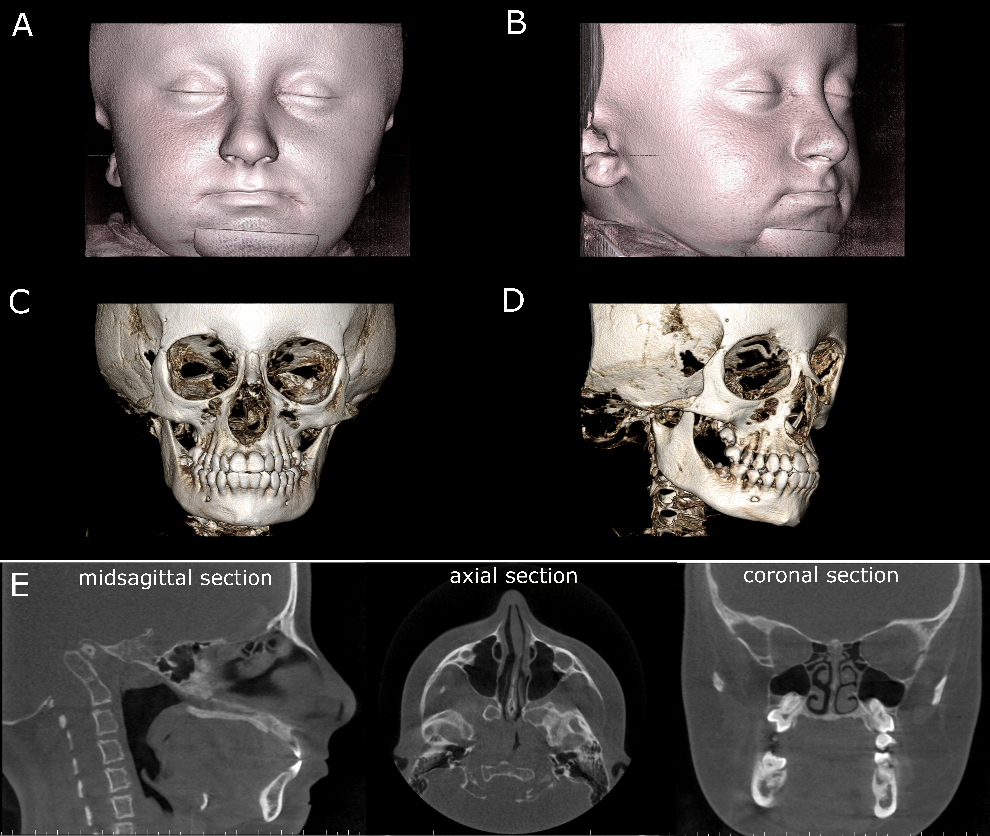


Fig. S2. Twin K. has less severe basilar invagination than her sister, but more pronounced extreme flattening of the cranial base angle. She also has a moderate nasal septum deviation and her pharyngeal airways volume is significantly lower than her sister’s volume (4 ml^3^ versus 12 ml^3^, see Fig. 2).
